# Tackling Non-Communicable Diseases In Low- and Middle-Income Countries: Is the Evidence from High-Income Countries All We Need?

**DOI:** 10.1371/journal.pmed.1001377

**Published:** 2013-01-29

**Authors:** Shah Ebrahim, Neil Pearce, Liam Smeeth, Juan P. Casas, Shabbar Jaffar, Peter Piot

**Affiliations:** 1 London School of Hygiene and Tropical Medicine, London, United Kingdom; 2 South Asia Network for Chronic Disease, Public Health Foundation of India, New Delhi, India; 3 Centre for Public Health Research, Massey University, Wellington, New Zealand; 4 Department of Epidemiology and Public Health, University College London, London, United Kingdom

## Abstract

Shah Ebrahim and colleagues argue that more research on non-communicable diseases (NCDs) in both high-income countries and low- and middle-income countries can result in mutual benefits and will help better address the growing burden of NCDs.

**Summary Points**

- Applied health research and development for non-communicable diseases (NCDs) in low- and middle-income countries (LMICs) is limited, and despite repeat calls for action, the NCD burden is increasing unchecked.
- NCD research in high-income countries (HICs) and LMICs can result in mutual advantages in the areas of replication and extending findings; discovering new causes of NCDs; studying health effects of exposures rare or ubiquitous in HICs; and exploring links between infectious diseases and NCDs.
- Different NCDs are at varying stages of needing research, policy development, and action. These stages range from not knowing the population burden of many NCDs to knowing all we need to take action.
- Changes in the global and national funding agendas will be required to strengthen the research and health system capacity for NCDs, which should reduce deaths and disability attributable to NCDs and yield economic dividends.

Non-communicable diseases (NCDs, e.g., cardiovascular disease, diabetes, cancers, chronic respiratory diseases, neurological diseases) have been *the* commonest cause of death and disability globally for at least the last three decades [1]. Even in sub-Saharan Africa, NCDs contribute a third of the disability-adjusted life year burden. However, research resources allocated to NCDs in low- and middle-income countries (LMICs) are trivial [2].

We analyse the interplay between applied health research in NCDs in high-income (HICs) and LMICs and demonstrate that there are opportunities for mutual advantages. We argue that different NCDs are at varying stages in a cycle of research, policy development, and action. The research and actions that are needed depend on the stages of this cycle.

## The Interplay between Research in HICs and LMICs

There is abundant information on the prevention and control of major NCDs from HICs, but little attention has been given to how research in LMICs can benefit HICs. Box 1 illustrates some of the forces arising in HICs, which are now having global impacts on NCDs. Research methods developed in HICs to study these forces are equally applicable to LMICs. In this section we explore this interplay.

**Box 1. Globalisation and NCDs**

- Tobacco
  - Aggressive marketing
  - Advertising, product placement
  - Political lobbying
  - Agricultural production
- Food availability and pricing
  - Trade agreements, corporate production, global distribution
  - Reduced taxes on unhealthy food imports
- Food preferences and ways of consuming
  - Pre-prepared and outside of home
  - Multimedia and marketing of western lifestyles and diets
  - Carbonated chilled sweetened drinks
- Physical activity
  - Promotion of car industry
  - Urban design
- Technologies
  - Communications (e.g., mHealth; eHealth)
  - Pharmaceuticals
  - Health care providers
- Social movements
  - Knowledge diffusion
  - Consumer influence
  - Patient interest groups
- Occupational exposures
  - Transfer of hazardous industries
  - Effects of occupational exposures in vulnerable populations

There are global benefits from repeating studies of “established” risk factors in LMICs. The first indication that there is no “safe” level of blood cholesterol came from research in China showing increased rates of coronary heart disease (CHD) even at low levels of blood cholesterol [3]. This finding spurred trials of statins among people with average cholesterol levels, leading to their widespread use regardless of blood cholesterol level for high risk individuals in HICs and LMICs [4].

Replicating health promotion trials for CHD in LMICs is sensible as rates are rising, health literacy is low, and there are strong views about these interventions [5],[6]. A recent large trial of health promotion in rural India demonstrated no effects on risk factor profiles or health knowledge [7]. These findings are disappointing, but may avoid wasteful investment in this particular approach in both HICs and LMICs.

The causes of many NCDs are unknown and therefore ways to prevent them remain elusive. Combined studies in LMICs and HICs may be more powerful, as there is often greater variation in exposure levels and marked differences in underlying confounders of risk factor–NCD associations. For example, breastfeeding appears to lead to lower blood pressure and body mass index in children in HICs, but no associations are found in LMICs, making it unlikely that the association in HICs is causal [8].

The upstream determinants of known causes of NCDs may differ between LMICs and HICs. High blood pressure, dyslipidaemia, and smoking are important proximal causes of cardiovascular disease globally, but their upstream causes reflect potentially modifiable social, fiscal, and legal environments that influence our behaviours and vary between HICs and LMICs [9]. Upstream determinants include economic [10], educational [11], occupational [12], agricultural [13], and trade [14] policies, all of which are adversely affecting risk factors globally.

Some exposures—for example, pesticides—do not occur widely or at high levels in HICs, making identification of potential health hazards difficult. Exposures that are orders of magnitudes higher occur in LMICs and enable harms to be identified, encouraging control of pesticides globally.

Forces that are near-ubiquitous in HICs (see Box 1) make it impossible to detect adverse effects through studies conducted in HICs [15],[16]. For example, asthma prevalence increases as countries become more economically productive and cleaner. The hygiene hypothesis suggests that lower childhood infection rates may “programme” the immune system, leading to asthma and allergy. The “hygiene hypothesis” cannot be tested only in HICs and requires global studies [17].

Strong associations have been reported between HIV/AIDS and cardiometabolic disorders [18]–[20], smoking and tuberculosis [21],[22], and diabetes and tuberculosis [23]. Cancers with an infectious aetiology are more common in LMICs and include gastric cancer (*Helicobacter pylori*), hepatocellular cancer (hepatatis B and C), and cervical cancer (human papilloma virus) [24]–[26]. Furthermore, the high burden of infectious diseases and associated chronic inflammation may exacerbate risks for some NCDs. The underlying mechanisms of these associations, new therapeutic targets, and opportunities to integrate communicable and NCDs in health sector reforms may be found by doing studies in LMICs [27].

Identifying the underlying forces that influence government and private sector decision-making on agriculture, trade, and the built environment requires research. Understanding how the tobacco industry, for example, operates in HICs helped shape the World Health Organization (WHO) Framework Convention on Tobacco Control [28], and led to effective counter-measures that are now being applied in LMICs. Similar research is needed in these other areas, particularly the food sector [29],[30]. Reliance on fiscal and legal interventions based on simple models examining the direct effects on consumption and other behaviours are becoming insufficient as there is growing understanding of the macroeconomic “ripple” effects of interventions across economic sectors and between countries [31].

New approaches to treatment of NCDs in LMICs are needed. The HIV/AIDS movement arising in HICs showed the importance of working with all stakeholders. The largest constituency affected by NCDs—elderly people—has been ignored in NCD discourses and alliances [32]. Elderly people constitute a major pressure group for achieving better, integrated, holistic services that respond to *all* their health needs.

Primary health care, supported by family and self-care, will form the backbone of cost-effective ways of treating and caring for NCDs and will require research to develop optimal care models [33]. Research on task shifting to non-medically qualified practitioners, low-cost near patient diagnostics, and self and family management are needed [34], and are also of relevance to HICs [35]. In addition, there is a great need to evaluate prevention interventions in different contexts in LMICs, given the current paucity of evidence. The results should be useful for informing prevention programmes with ethnic minorities in HICs.

### The Political Need for Research

The inexorable rise of NCDs in LMICs has been left unchecked for two decades with major economic consequences and avoidable loss of lives. The current situation is unduly influenced by economic and commercial interests that negate the importance of NCDs [36]. Global and local research, particularly if it can be conducted in parallel in HICs and LMICs, can provide powerful arguments for the need to act globally, as envisaged in the 2011 United Nations high-level meeting on NCD prevention and control [37].

## Situations: Research and Action

Research agendas for LMICs have been published recently [38]–[41] proposing what needs to be done. We are concerned that “action” only may seem to be preferable to “research” in LMICs to deal with the rising NCD burden. Research and action are not opposite extremes of a continuum but responses that arise depending on specific situations, which are summarized in Box 2.

**Box 2. Situations: Research and Action Examples**

*Situation 1: We don't even know the population burden of many NCDs*

We lack data on the burden of disease even for common conditions such as asthma and epilepsy. Adding NCD modules to existing health and demographic surveillance systems that provide burden of disease estimates for maternal and child health is underway and will provide additional sources of robust data [60].

*Situation 2: We know the population burden but we don't know the causes*

Understanding the “upstream” causes of NCDs can make use of natural experiments such as the introduction of urban mass transport systems on physical activity [61]; social and economic change on risk factors [62]; and rural development strategies (e.g., new roads, employment schemes) on obesity and diabetes. The Prospective Urban Rural Epidemiology [63] study runs in 600 communities in 17 countries to examine the impact of urbanisation on health.

*Situation 3: We know the causes but we need more research into methods of prevention*

Occupational exposures make a substantial contribution to NCD in industrialised countries, but their relevance in LMICs has not been assessed [64]. This is of particular concern given that many hazardous industries are situated in LMICs [65].

For example, in India's asbestos industry, health risks are discounted: “That lung cancer deaths have been caused by asbestos fibre has not been proved in India,” argues John Nicodemus, executive director of the Asbestos Cement Products Manufacturers' Association, a New Delhi–based industry organization [66].

*Situation 4: We need research to improve patient treatment and care*

Management of NCDs through mHealth technology, task shifting from doctors to other health workers, and self-management all require robust evaluation. They may also be highly relevant to cost-constrained health services in HICs.

*Situation 5: “We know what is needed—but not how to implement it”*

Salt restriction lowers blood pressure but is difficult for individuals to do as much dietary salt is hidden in processed foods. In the UK, bread manufacturers have voluntarily reduced the salt content of bread slowly, which should result in reductions in population levels of blood pressure [67].

First, the population burden of many NCDs is not even known. Recent global burden of disease studies have produced modeled estimates derived from existing, but patchy, data of common risk factors trends to fill the information gap [42]–[45]. Existing surveillance and monitoring systems require expanding to include the major NCDs and risk factors to improve estimates of burden and monitor trends in LMICs, as has been done for asthma [46].

Second, while the population burden of some diseases is known, the causes aren't. Rapid socioeconomic growth in many LMICs, alongside the severe economic crisis affecting HICs, and growing migration and urbanisation are generating “natural experiments” that will allow investigation of the upstream determinants of common risk factors for NCDs. For example, a recent study examining the association of unemployment in an economic recession and the increasing number of suicides implicated a lack of social protection systems in the United States (compared with other European countries) as a causal factor [47].

Third, when causes are known there still needs to be more research into methods of prevention. Tobacco control topped the UN high-level meeting's priority list for action [37]. The total global population covered by comprehensive smoke-free laws increased from 3.1% in 2007 to 5.4% in 2008, providing protection for an additional 154 million persons [48]. While this is a big relative improvement in a short period of time, it is clear that current strategies are failing the remaining 95% of the global population, and tobacco use is still increasing globally.

Fourth, research is needed to improve patient treatment and care. In parallel with prevention, improved patient management is essential [49]. Health services research, well developed in HICs, is needed to identify cost-effective treatments, and implementation research is then required to get research findings into practice and improve quality of health care [50]. The potential for improving NCD health care through health services research is huge: for example, eHealth; non-invasive imaging to aid diagnosis; and integrated patient health records. Many of these developments, pioneered in LMICs and evaluated collaboratively using robust methods, will likely yield global benefits through reverse innovation [51].

Finally, a situation where what is needed is known, but not how to implement it. There are some NCDs for which it can be argued that sufficient knowledge is available to act now. For example, five priority interventions—tobacco control, salt reduction, improved diets and physical activity, reduction of hazardous alcohol intake, and access to essential drugs and technologies—were recently defined as requiring “leadership, prevention, treatment, international cooperation, monitoring and accountability” [52]. Experiences from both HICs and LMICs will be relevant in finding the best ways forward.

## Economics of NCDs and Funding Research For NCDs

The economic consequences of NCDs are large and have been well documented. Estimates of the lost output attributable to NCDs amount to trillions of dollars a year [53]; the costs of simple effective interventions are measured in millions of dollars [54]. Research investments are now required urgently to fill the implementation gap between what works and achieving health gain in practice. The UN high-level meeting on NCDs formally acknowledged that resources devoted to combating NCDs are not commensurate with the magnitude of the problem [37]; the meeting noted that domestic, bilateral, regional, and multilateral channels of funding will be required.

Most health research funding is spent in HICs, but the greatest need, both in scientific and public health terms, is in the rest of the world [55]. Previous calls to action on NCDs over the last decade have had some impact on funding [54], which may generate further enthusiasm for funding research in LMICs.

While most LMICs do have a budget for NCD-related work [56], there is no room to innovate and evaluate strategies for NCDs. Much of the research and development relevant to implementation is country-specific and requires national funding. The WHO has recommended that the extra resources needed could come from increasing efficiency of revenue collection; improving access to social health insurance; increased tobacco and alcohol taxes; and including NCDs as a priority for official development assistance [57]. A further need is to develop capacity to conduct applied NCD health research. This is limited in most LMICs. Training and partnerships with experienced NCD researchers and institutions should be a high priority for development programmes.

The time has now come for all health-related research and development funders—global, regional, and national— to acknowledge the existence of NCDs and rise to the challenges they present. For example, the United Kingdom's Department for International Development has identified the importance of NCDs in contributing to poverty and has initiated a mental health programme in several LMICs. Hopefully other programmes will follow [58]. A first step would be for global and bilateral agencies, major national health research councils, and charities to publish their spending by disease categories to track their contribution in meeting the World Health Assembly's recent NCD targets of a 25% reduction in NCD mortality by 2025 [59].

## Conclusions

There are unique opportunities for answering critical research questions about NCDs in LMICs (see Box 3). The list reflects our experiences and disciplinary perspectives from public health, epidemiology, primary care, and health policy. Where to begin will depend on the scientific capacity to deliver, national priorities, and the funds available. The important point is to make a start somewhere. The 2011 UN high-level meeting provided a strong context for research and action. A major shift in attitudes from knowing what needs to be done towards using research to prioritise, evaluate, monitor and, incrementally, improve health outcomes is urgently needed. Action and research are required: they are intimately entwined and the balance between them will depend on the situation and the health problem.

**Box 3. A Proposed Research Agenda for NCDs in Low- and Middle-Income Countries**

- Measuring the burden of NCDs, mental health, and injuries
  - Improving synthetic methods
  - Integrating and improving quality of health information systems
- Causal inference:
  - “Upstream” causes of common NCD risk factor distributions
  - Occupational exposures and NCDs
- Genetic variation
  - Mendelian randomisation approaches to identifying environmental determinants of NCDs
  - Pharmacogenetics for stratified medicine to minimise risks and maximise benefits of treatments
- Urbanisation/migration
  - Influence of urbanisation on lifestyles
  - Health impacts of urban and rural development programmes
- Prevention and control of NCDs
  - Cost-effectiveness studies of preventive interventions targeted at individuals
  - Cost-effectiveness studies of fiscal and legal means of health protection
  - Developing and promoting healthier models of food production, marketing, and consumption
- Health systems research
  - Strengthening of primary care services
  - Health care financing for universal primary care coverage
  - Task-shifting, family and self-care, and eHealth for NCD prevention and care
- Policy research
  - Evaluation of health impacts of public policies on food security, trade, agriculture, and rural/urban development
- Implementation research
  - Use of health technology assessment and audit to improve quality of health care
  - Examination of facilitators and barriers to establishing cross-sectoral working for health

## Abbreviations

HIC: high-income country
LMIC: low- and middle-income country
NCD: non-communicable disease
UN: United Nations
WHO: World Health Organization
